# Knowledge of Cervical Cancer and Acceptability of Prevention Strategies Among Human Papillomavirus-Vaccinated and Human Papillomavirus-Unvaccinated Adolescent Women in Eldoret, Kenya

**DOI:** 10.1089/biores.2019.0007

**Published:** 2019-08-20

**Authors:** Anisa Mburu, Peter Itsura, Hillary Mabeya, Alice Kaaria, Darron R. Brown

**Affiliations:** ^1^ Department of Reproductive Health, Moi Teaching and Referral Hospital, Eldoret, Kenya.; ^2^ Department of Reproductive Health, Moi University, Eldoret, Kenya.; ^3^ Reproductive Health Services, Nairobi, Kenya.; ^4^ Department of Medicine, Indiana University School of Medicine, Indianapolis, Indiana.

**Keywords:** acceptability, adolescents, HPV vaccine, Kenya, knowledge

## Abstract

Cervical cancer is a critical public health concern in sub-Saharan Africa. Adolescents are key targets in primary prevention strategies. Following a human papillomavirus (HPV) vaccination initiative (*Gardasil*) in Eldoret, Kenya, the knowledge and source of information of cervical cancer and acceptance of prevention strategies among vaccinated and unvaccinated adolescents were evaluated. A cross-sectional comparative study enrolled 60 vaccinated and 120 unvaccinated adolescent women. Institutional ethical approval was obtained and signed consent was obtained from the parents. Data collection was performed using interviewer-administered questionnaires derived from factual statements based on information from print material used for community sensitization on cervical cancer. The median age of the participants was 14.0 years (interquartile range [IQR] = 13.0–15.0). Of 60 vaccinated adolescents, 56 (93.3%) had heard of the HPV vaccine compared with 6 (5%) of unvaccinated participants (*p* < 0.001). Of 60 vaccinated participants, 58 (96.7%) had heard of cervical cancer compared with 61 (50.8%) unvaccinated participants (*p* < 0.001). Both cohorts identified the school as the main source of information for cervical cancer. The two groups also showed similarity in their selection of cervical cancer prevention strategies acceptable to them such as delaying sexual debut, limiting number of sexual partners, and use of condoms for protection against sexually transmitted infections. Of 120 unvaccinated participants, 63.7% expressed willingness to be vaccinated. Exposure to the HPV vaccine was associated with a higher knowledge of cervical cancer. The adolescents predominantly rely on the school for health information. Both cohorts of adolescents showed remarkable acceptability for cervical cancer prevention strategies.

## Introduction

Cancer of the cervix has ascended the oncological ladder to be the primary fatal gynecological cancer in developing countries.^1^ In 2012, 270,000 women died from cervical cancer with 85% from low- and middle-income countries. There were 530,000 new cases in 2012 worldwide.^2^ Cancer of the cervix is increasingly becoming a public health concern in sub-Saharan Africa; it ranks as the first cause of female cancers in Kenya, and is the leading cause of cancer deaths/mortality among all cancers in women.^1,2^ Annually, the estimated number of cervical cancer cases in Kenya is 5250, whereas the mortality is 3286.^3^

Human papillomavirus (HPV), a sexually transmitted virus, is the causative agent of cervical cancer with the highly oncogenic HPV types 16 and 18 present in >90% of invasive cervical carcinoma specimen in Western Kenya.^4,5^ In sexually active adolescents, HPV prevalence is high with 50–80% of adolescents having active infections within 12 months of sexual debut.^6^

It follows that the contemporary approach is shifting toward primary prevention strategies with principal emphasis on adolescents to foster early behavioral modification including but not limited to delaying sexual debut, restricting number of sexual partners, consistent correct condom use, and not engaging in smoking.^7^ Recent scientific advances also evidenced the inception of HPV vaccines that have proved effective in the prevention of HPV infection and associated disease with many nations incorporating it in their national adolescent vaccination schedule.

The success of these primary cervical cancer prevention strategies is related to the level of awareness and knowledge regarding various aspects of the disease and vaccine.^8^ A study in Kenya showed that most Kenyan women had very little knowledge about HPV and HPV vaccination. Some women confused HPV with HIV or hepatitis B. They were willing to let their daughters be vaccinated if it meant prophylaxis against cancer; however, they stated that they would prefer a more inexpensive option with fewer dosages.^9^

However, there are a dearth of data on the level of awareness of cervical cancer among Kenyan adolescents and their acceptance of cervical cancer prevention strategies. Therefore, a study was conducted to compare the knowledge and source of information of cervical cancer and acceptability of prevention strategies among vaccinated and unvaccinated adolescent girls after a HPV vaccination initiative in Eldoret, Kenya that was carried out by Gardasil Access Program, under Merck and Co., Inc., in 2012 to 2013.

## Methods

### Cohort enrollment

This cross-sectional comparative study was carried out between May and October 2016 in six randomly selected public schools that had participated in the Gardasil Access Program's hospital-based HPV vaccination initiative in Eldoret, Kenya 3 years before this study was conducted. Using lists from each school with the names of the vaccinated adolescents, we randomly selected a total of 60 vaccinated adolescents by proportionate allocation from 3 of the schools. For the comparative cohort, we selected 120 unvaccinated adolescents from the remaining 3 schools also by proportionate allocation. The eligibility criteria included being 12–18 years of age at the time of enrollment (3 years after the vaccination initiative) and being female gender; this is in accordance to the World Health Organization definition of an adolescent (10–19 years).

### Ethical issues

The Moi University and Moi Teaching and Referral Hospital Institutional Research and Ethics Committee in Eldoret, Kenya approved the study protocol. As all participants were minors (<18 years) in accordance to the Kenyan constitution, written informed consent was required from each parent/guardian with subsequent participant informed assent.

### Study procedures

After enrolment, the adolescents had face-to-face interviews with the principal investigator and trained research assistants in classrooms or under tree shades depending on the participant's preference in a bid to create an informal set up. The interviews were carried out in English, as it is the primary language for communication at all schools in Eldoret, covering demographic details, questions on HPV transmission, and identification of cervical cancer symptoms and treatment options. The questions were based on print material used to disseminate information about cervical cancer. Each question had three choices: “Yes,” “No,” and “Don't know” (Supplementary Table S1). The adolescents were also asked to select cervical cancer screening modalities that they found acceptable to them with future intentions for uptake.

### Statistical analysis

Categorical variables were summarized using frequencies and the corresponding percentages. Continuous variables were summarized using mean and the corresponding standard deviation if the Gaussian assumptions were holding. The median and the corresponding interquartile range (IQR) whenever the Gaussian assumptions were violated. Gaussian assumptions were assessed using Shapiro–Wilk test, the normal probability plots and histograms.

Proportions were compared using Pearson's chi-squared test. However, whenever the chi-squared assumptions were violated, Fisher's exact test was used. Normally distributed continuous variables were compared between two levels of a categorical variable using independent sample *t*-test or one-way analysis of variance test between more than two levels of a categorical variable. The two-sample Wilcoxon rank sum test was used to compare two continuous variables whenever the Gaussian assumptions were violated. The knowledge score was compared between the vaccinated and the unvaccinated using a linear regression model adjusting for potential confounding variables. This was reported by the regression estimates and the corresponding 95% confidence intervals (CIs). The statistical package used for data analysis was carried out using R: A Language and Environment for Statistical Computing for summarizing and comparing the variables for significant statistical inference.^10^

## Results

### Demographics

The median age of all 180 participants was 14.0 years (IQR = 13.0–15.0) with a range of 12.0–18.0 years. Eighteen of 180 girls (10%) were in grade 6 (12–13 years of age), 56 of 180 girls (31.1%) were in grade 7 (13–14 years of age), and 106 of 180 girls (58.9%) were in grade 8 (14–15 years of age). No vaccinated girls were in grade 6. Forty-nine of the 60 (81.7%) vaccinated adolescents were in the eighth grade with the mean age for vaccination being 14 years.

### Knowledge

Of the vaccinated adolescents, 96.7% (58/60) had heard of cervical cancer as compared with 50.8% (61/120) of the unvaccinated adolescents (*p* < 0.001). A majority of the vaccinated adolescents (93.3%, 56/60) had heard of the HPV vaccine, whereas only 5% (6/120) of the unvaccinated adolescents had heard of the vaccine (*p* > 0.001). HPV was correctly identified as a sexually transmitted virus by 41.7% (25/60) vaccinated adolescents but only by 5% (6/120) unvaccinated adolescents (*p* < 0.001).

In terms of knowledge of risk factors for cervical cancer, 93.3% (56/60) vaccinated girls knew that an early sexual debut was a predisposing factor for cervical cancer, compared with 70.8% (85/120) of unvaccinated girls (*p* = 0.001). There was no statistically significant difference in knowledge between the vaccinated and unvaccinated adolescents in selecting other risk factors for cervical cancer such as smoking and having multiple sexual partners (Table 1).

**Table 1.** Comparison of Knowledge of Cervical Cancer, Human Papillomavirus and its Risk Factors

Variable	Vaccinated		p^a^
	No (*N* = 120)	Yes (*N* = 60)	
	Participants, *n* (%)		
General knowledge			
Heard of cervical cancer (yes)	61 (50.8)	58 (96.7)	<0.001
Heard of HPV vaccine (yes)	6 (5.0)	56 (93.3)	<0.001
HPV transmission			
Sexual contact (correctly responded yes)	6 (5.0)	25 (41.7)	<0.001
Blood transfusion (incorrectly responded yes)	6 (5.0)	8 (13.3)	0.074
Don't know	108 (90.0)	27 (45.0)	<0.001
Cervical cancer risk factors^b^			
Early sexual debut (correctly responded yes)	85 (70.8)	56 (93.3)	0.001
Smoking (correctly responded yes)	62 (51.7)	33 (55.0)	0.792
Multiple sexual partners (correctly responded yes)	90 (75.0)	49 (81.7)	0.414
Male partner with multiple partners (correctly responded yes)	101 (84.2)	49 (81.7)	0.832

^a^ Significance is at *p* ≤ 0.05.

^b^ Correctly answered yes.

There was no significant difference between vaccinated and unvaccinated participants in awareness that cervical cancer is minimally symptomatic in its early stages (Table 2). A majority of the vaccinated adolescents correctly identified chemotherapy, radiation, or surgery as viable treatment modalities for cervical cancer (*p* < 0.001) (Table 2).

**Table 2.** Comparison of Knowledge of Symptom Profile, Treatment, and Screening Modalities for Cervical Cancer

Variable	Vaccinated		p^a^
	No (*N* = 120)	Yes (*N* = 60)	
	Participants, *n* (%)		
Correct responses for symptom profile of cervical cancer			
Early stage (correctly responded yes)			
Few symptoms	10 (8.3)	2 (3.3)	0.342
Late stage (correctly responded yes)			
Lower abdominal pain	69 (57.5)	47 (78.3)	0.010
Vaginal bleeding	73 (60.8)	38 (63.3)	0.871
Vaginal discharge	75 (62.5)	35 (58.3)	0.705
Anemia	43 (35.8)	26 (43.3)	0.416
Postcoital bleeding	61 (50.8)	37 (61.7)	0.224
Urine and fecal incontinence	38 (31.7)	30 (50.0)	0.026
Weakness	78 (65.0)	45 (75.0)	0.234
Correct responses for treatment options			
Chemotherapy (correctly responded yes)	22 (18.3)	46 (76.7)	<0.001
Radiation (correctly responded yes)	9 (7.5)	29 (48.3)	<0.001
Surgery (correctly responded yes)	18 (15.0)	36 (60.0)	<0.001
Don't know	79 (65.8)	5 (8.3)	<0.001
Correct responses for screening options			
Blood tests (correctly responded no)	40 (33.3)	32 (53.3)	0.015
Pap smear (correctly responded yes)	11 (9.2)	10 (16.7)	0.218
VIA (correctly responded yes)	13 (10.8)	7 (11.7)	>0.999
Don't know	60 (50.0)	21 (35.0)	0.080

^a^ Significance is at *p* ≤ 0.05.

VIA, visual inspection with acetic acid.

Regarding knowledge of screening options, 35% (21/60) from the vaccinated cohort and 50% (60/120) from the unvaccinated cohort indicated that they did not know the cervical cancer screening modalities available but there was no significant difference between the two groups. For the adolescents who correctly selected Pap smear and visual inspection with acetic acid as screening modalities, 28.4% (17/60) were from the vaccinated group, whereas 20% (24/120) were from the unvaccinated group, also with no significant difference between the two groups. Blood tests were ruled out as cervical cancer screening modalities by 53.3% (32/60) of the vaccinated adolescents and by 33.3% (40/120) of unvaccinated adolescent girls (*p* = 0.015) (Table 2).

There was no statistical difference between the vaccinated and unvaccinated adolescents in their opinion that cervical cancer was preventable.

In multivariate analysis, adolescents who were vaccinated were more likely to be knowledgeable about cervical cancer than those in the unvaccinated counterparts, regardless of the current grade of school (adjusted odds ratio = 14.4; 95% CI = 12.2–16.7) (Table 3).

**Table 3.** Predictors of Knowledge of Cervical Cancer Derived by Logistics Regression Analysis

Variable	Unadjusted	Adjusted
	OR^a^ (95% CI)	OR^a^ (95% CI)
Vaccinated	14.7 (12.6–16.8)	14.4 (12.2–16.7)^b^
Grade 8	5.3 (2.5–8.1)	0.7 (−1.4 to 2.9)

^a^ OR: subject to logistic regression, only a positive vaccination status was the true predictor of knowledge of cervical cancer, with being a student in grade 8 not increasing the chance of being knowledgeable.

^b^ Significance is at *p* ≤ 0.05.

CI, confidence interval; OR, odds ratio.

### Source of information

The potential sources of information the adolescents relied on to obtain information about cervical cancer were categorized to include the school, health care workers, social media, and community outreach programs. Both cohorts selected the school as the main source of information for cervical cancer (Table 4). Despite the vaccination process being hospital based, only 22.4% (13/60) of the vaccinated adolescents reported having heard about cervical cancer from a doctor.

**Table 4.** Comparison of Source of Information for Cervical Cancer Among Participants Unvaccinated and Vaccinated Against Human Papillomavirus

Variable	Vaccinated		p
	No (*N* = 120)	Yes (*N* = 60)	
	Participants, *n* (%)		
Source of information^a^			
Community outreach program	2 (3.3)	4 (6.9)	0.431
Doctor	5 (8.2)	13 (22.4)	0.040
Social media	19 (31.1)	13 (22.4)	0.308
Family member/friend suffered from it	11 (18.0)	11 (19.0)	>0.999
School Health Talk	22 (36.1)	20 (34.5)	>0.999

Significance is at *p* ≤ 0.05.

^a^ Number of participants selecting the option best applicable to them.

### Acceptance of cervical cancer prevention strategies

An assessment of the individual perceived risk of infection by HPV showed that 53.3% (32/60) of the vaccinated participants reported a higher sense of self-perceived risk compared with 22.5% (27/120) of the unvaccinated cohort (*p* < 0.001) (Table 5).

**Table 5.** Comparison of Acceptability of Prevention Strategies for Cervical Cancer Among Participants Unvaccinated or Vaccinated Against Human Papillomavirus

Variable^a^	Vaccinated		p
	No (*N* = 120)	Yes (*N* = 60)	
	Participants, *n* (%)		
Self-perceived risk of HPV infection	27 (22.5)	32 (53.3)	<0.001
Acceptable age of sexual debut (years)			
10–20	2 (1.7)	0(0.0)	0.553
21–30	68 (56.7)	32 (53.3)	0.791
≥31	50 (41.7)	28 (46.7)	0.632
Acceptable frequency of using condom (*n* = 178)^b^			
Always	87 (73.7)	41 (68.3)	0.561
Sometimes	21 (17.8)	12 (20.0)	0.878
Never	10 (8.5)	7 (11.7)	0.678
Would agree to receive HPV vaccine (*n* = 118)^c^	75 (63.6)		
Would agree to be screened for cervical cancer	102 (85.0)	52 (86.7)	0.940
Vaccination protects you from all STI so no need for safe sex practices^d^ (*n* = 60)		11 (18.3)	

Significance is at *p* ≤ 0.05.

^a^ Number of participants selecting the option best acceptable to them.

^b^ Use of the male condom. Figures do not add up to 180 as 2 girls did not respond.

^c^ Figures do not add up to 120 as 2 girls did not respond.

^d^ Based on the opinion of only the girls vaccinated on the kind of protection they now have through vaccination.

STI, sexually transmitted infection.

A 21- to 30-year-old age range was selected by both the vaccinated and unvaccinated cohorts as being the most preferable for initiating a sexual debut (*p* = 0.791) rather than the 10–20 years age range or >31 years of age.

A high frequency of use of male condoms was also deemed acceptable by both the vaccinated (68.3%; 41/60) and the unvaccinated (73.7%; 87/120) adolescents.

Seventy-five of 118 unvaccinated adolescents (63.6%) would accept the HPV vaccine with a majority of those who would not accept it (43.2%, 16/37), reporting it was because of the lack of knowledge on what it is.

Among those vaccinated, 18.3% (11/60) thought that because they were vaccinated, they were protected from all sexually transmitted infections (STIs) and so would not need to engage in safe sex practices.

A bivariate analysis tested for association between uptake of cervical cancer screening with relation to the vaccination status. As given in Table 5, similar proportions of participants among those who had been vaccinated (86.7%, 52/60) and those unvaccinated against HPV (85%, 102/120) would accept cervical cancer screening (*p* = 0.940).

## Discussion

### Demographics

Numerous studies have been carried out locally and internationally with regard to knowledge and attitudes toward HPV and HPV vaccine.^10^ The adolescent women in this study were matched for age, sex, and socioeconomic status by selection of schools in the same low resource setting. The defining element was vaccination status. The vaccinated adolescents were predominantly in grade 8 as they had been vaccinated 3 years before this study.

### Knowledge

It was anticipated that the vaccinated adolescents would be more knowledgeable about cervical cancer and thus perceive themselves to be at a higher risk of HPV infection.^12,13^ In this study population, the adolescents in both groups perceived themselves to be at a higher risk of acquiring the virus but this could be attributed to the very low knowledge they had on the transmission of HPV, or to undisclosed sexual activity. Adolescents from both the vaccinated and unvaccinated cohort could identify some of the risk factors for acquisition of HPV, including an early sexual debut, multiple sexual partners, or having a sexual partner with multiple sexual partners and smoking. Knowledge of treatment and screening modalities for cervical cancer was low in both groups as seen in other adolescent populations where there is a low level of knowledge of HPV, its transmission, causes, and prevention strategies for cervical cancer among adolescents.^14–16^ This shows that as much as a positive vaccination status may be associated with better knowledge of cervical cancer, it does not afford sufficient awareness of the same if education offered during vaccination is not comprehensive and sufficient.

Previous studies have shown concern among parents that HPV vaccination may give the adolescent a perception of being safeguarded from all sexually transmitted illnesses and thus propelling them into promiscuity.^17,18^ Neither group in this study believed that the vaccine would prevent all STIs, similar to a previous study^19^ that strove to dispel this myth by exploring the association between getting the vaccine and engaging in risky sexual behavior, finding no such evidence. Studies looking for risky sexual behaviors after HPV vaccination found that vaccination was a good opportunity to reiterate the need for safe sex practices among adolescents and young women.^20^ In Western Uganda,^21^ it was established that HPV vaccination, knowledge and perceived sexual risk did not predict sexual behavior intentions, and with high parental involvement and communication, sexual debut could be delayed. The importance of this lies in the fact that early sexual debut and early age at first pregnancy have been linked to the development of cervical cancer later in life.^22^

The adolescents reported a high potential for use of condoms primarily as a protective measure against pregnancy and HIV/AIDS. Several studies locally and internationally recount several barriers for adolescents to accessing condoms such as unavailability of youth-friendly facilities providing free condoms, judgmental health practitioners, and social disapproval; common barriers experienced by adolescents in low- and middle-income countries.^23^ A study in Sweden showed that a school-based program promoting condom use increased the uptake of condoms for HPV prevention among adolescents.^24^

### Source of information

There are several sources of information for HPV and cervical cancer such as social media, radio/television advertisements, print material, community outreach health talks, and information garnered from a health professional at a hospital to name but a few.

From the data accrued from the interviews, the adolescent girls in this study showed immense reliance on their teachers for information on health. This is in line with the surmise of Masika et al.^25^ that the empowerment of teachers would be a more feasible way to increase uptake of the vaccine through their dissemination of information on the vaccine and cervical cancer in a low resource setting such as in Eldoret, Kenya.

Similarly, a previous study in this region^26^ saw the need for a collaborative effort between health workers and teachers to provide correct information while tackling stigmatism and myths that are a hindrance to the primary prevention of cervical cancer.

It is clear that adolescents need exposure to other avenues of information disbursement and need to be given access to other sources at their level. For instance, the Cancer Registry of Norway developed an application called FightHPV™, an interactive game that can be downloaded on various android and Apple devices^27^ by adolescents and anyone with limited knowledge of HPV and cervical cancer to learn and understand the disease and the ways to protect oneself.

### Acceptability

The unvaccinated group showed great willingness to be vaccinated, which was higher compared with other studies such as that of Moroccan adolescents, which revealed that only 27% (282/1044) of participants were willing to accept HPV vaccination.^28^

The acceptability of screening for cervical cancer was remarkably high in both cohorts of adolescents despite their minimal collective knowledge of screening modalities available. This was higher than most studies where the adolescents showed very low levels of desire to be screened.^13,15^ This phenomenon could be explained by the possible exposure to community-based messaging on cervical cancer in this region urging women to go for screening thus making the adolescents realize their own susceptibility to cervical cancer.

### Limitations

Because of the univariate results, the study was not powered to conduct a logistic regression that sufficiently shows association and predictors. Another potential limitation is that the informal interview settings utilized may have permitted a social desirability bias. However, during the pilot phase of the project, it was noted that the participants had trouble understanding the implication of some medical terms despite attempts to simplify the language. With an interviewer administering where the participants could ask for clarification, ease in responding was noted and thus this was the mode employed.

## Conclusion

Receiving the HPV vaccine was associated with a higher knowledge of cervical cancer.

The adolescent women in this study predominantly rely on their school for health information. Other sources of health information like interactive social media need to be availed to adolescents. Both cohorts of adolescents showed remarkable acceptability for cervical cancer prevention strategies. There is need to increase exposure to the vaccine that may also aid in improving knowledge of cervical cancer. Prevention strategies should be made more accessible to the adolescents to improve acceptability.

## Implications and Contributions

There is high morbidity and mortality from cervical cancer in sub-Saharan Africa. Thus the need to aggressively involve adolescent women in primary prevention strategies such as increasing HPV vaccination and behavioral modifications like condom use to prevent sexually transmitted diseases, limiting number of sexual partners, and regular screening.

## Authors' Contribution

A.M., conception and design, development of methodology, acquisition of data, analysis and interpretation of data, writing of article. P.I., project supervision, review, and revising of data critically for important intellectual content. H.M., project supervision, review, and revising of data critically for important intellectual content. A.K., concept design, review, and revising of data critically for important intellectual content. D.R.B., review and revising of data critically for important intellectual content, final approval of the version to be published.

## Supplementary Material

Supplemental data

## Abbreviations Used

CI: confidence interval
HPV: human papillomavirus
IQR: interquartile range
OR: odds ratio
STI: sexually transmitted infection
