# Supplementary material for: Knowledge of Cervical Cancer and Acceptability of Prevention Strategies Among Human Papillomavirus-Vaccinated and Human Papillomavirus-Unvaccinated Adolescent Women in Eldoret, Kenya
Source: Biores Open Access. 2019 Aug 20;8(1):139–45. doi: 10.1089/biores.2019.0007 (PMC6715544; doi:10.1089/biores.2019.0007)
Supplement: Supplemental data [file Supp_Table1.pdf]

Supplementary Data

Supplementary Table S1. Statements Used to Assess Knowledge

1	Have you ever heard of cervical cancer?
2	Have you ever heard of the HPV vaccine?
3	How is HPV transmitted?
4	Do you think having sex at an early age is a risk factor for cervical cancer?
5	Do you think smoking is a risk factor for cervical cancer?
6	Do you think having many sexual partners is a risk factor for cervical cancer?
7	Do you think having a male partner who has many sexual partners is a risk for cervical cancer?
8	A person suffering from early cervical cancer will have the following signs and symptoms
	(tick √ for Yes and cross X for No)
	Rare
9	A person suffering from late cervical cancer will have the following signs and symptoms
	(tick √ for Yes and cross X for No)
	Lower abdominal pain
	Vaginal bleeding
	Vaginal discharge
	Anemia
	Postcoital bleeding
	Involuntary loss of urine and/or feces through the vagina
	Weakness
	Weight loss
10	What are the treatment modalities available for cervical cancer?
	(tick √ for Yes and cross X for No)
	Antibiotics
	Chemotherapy
	Radiation
	Surgery
	I do not know
11	What are the screening modalities used to detect early cervical cancer?
	Blood tests
	Pap smear
	VIA/VILLI
	I don't know
12	Can cervical cancer be prevented?
13	Do you think you are at risk of getting HPV infection?
14	What do you think is the best age to be to start having sex?
	10–20
	20–30
	Above 30 years
15	How often do you think someone use male condom during intercourse?
	Sometimes Always Never
